# Engineering of near IR fluorescent albumin nanoparticles for in vivo detection of colon cancer

**DOI:** 10.1186/1477-3155-10-36

**Published:** 2012-08-14

**Authors:** Sarit Cohen, Shlomo Margel

**Affiliations:** 1 The Institute of Nanotechnology and Advanced Materials, Department of Chemistry, Bar-Ilan University, Ramat-Gan 52900, Israel

**Keywords:** HSA nanoparticles, Fluorescent nanoparticles, NIR fluorescence, Optical imaging, Colon cancer

## Abstract

**Background:**

The use of near-infrared (NIR) fluorescence imaging techniques has gained great interest for early detection of cancer because water and other intrinsic biomolecules display negligible absorption or autofluorescence in this region. Novel fluorescent nanoparticles with potential to improve neoplasm detection sensitivity may prove to be a valuable tool in early detection of colon tumors.

**Methods:**

The present study describes the synthesis and use of NIR fluorescent albumin nanoparticles as a diagnostic tool for detection of colon cancer. These fluorescent nanoparticles were prepared by a precipitation process of human serum albumin (HSA) in aqueous solution in the presence of a carboxylic acid derivative of the NIR dye IR-783 (CANIR). Tumor-targeting ligands such as peanut agglutinin (PNA), anti-carcinoembryonic antigen antibodies (anti-CEA) and tumor associated glycoprotein-72 monoclonal antibodies (anti-TAG-72) were covalently conjugated to the albumin nanoparticles via the surface carboxylate groups by using the carbodiimide activation method.

**Results and discussion:**

Leakage of the encapsulated dye into PBS containing 4% HSA or human bowel juice was not detected. This study also demonstrates that the encapsulation of the NIR fluorescent dye within the HSA nanoparticles reduces the photobleaching of the dye significantly. Specific colon tumor detection in a mouse model was demonstrated for PNA, anti-CEA and anti-TAG-72 conjugated NIR fluorescent HSA nanoparticles. These bioactive NIR fluorescent albumin nanoparticles also detected invisible tumors that were revealed as pathological only subsequent to histological analysis.

**Conclusions:**

These results may suggest a significant advantage of NIR fluorescence imaging using NIR fluorescent nanoparticles over regular colonoscopy. In future work we plan to broaden this study by encapsulating cancer drugs, such as paclitaxel and doxorubicin, within these biodegradable NIR fluorescent HSA nanoparticles, in order to use them for both detection as well as therapy of colon cancer and others.

## Background

Early detection of adenomatous colonic polyps is a major concern in the prevention of colon cancer [1,2]. Colorectal cancer can largely be prevented by the early detection and removal of adenomatous polyps. A variety of colorectal cancer screening modalities are available, including stool-based tests and radiological and endoscopic examinations of the colon [3,4]. These methods are considered to be either lacking in sensitivity or invasive, and colon cancer continues to be a major cause of death in the western world. Novel fluorescent nanoparticles with potential to improve neoplasm detection sensitivity may prove to be a valuable tool in early detection of colon tumors.

The key to effective specific imaging in deep tissues is the use of NIR light [5]. Absorption, light scattering and autofluorescence are limitations to fluorescence imaging that are significantly reduced in the NIR region (approximately 700 to 1000 nm). Biological chromophores, particularly hemoglobin, strongly absorb visible light, thereby limiting the depth of penetration at shorter wavelengths to a few millimeters. Other biological compounds such as water and lipids strongly absorb light in the infrared region. In addition, the decreased light scattering and autofluorescence in the NIR region provide the non-fluorescent background necessary for optimal detection of a fluorophore within the surgical field [6,7]. Nanoparticle-based NIR probes have been shown to have significant advantages over free organic NIR dyes such as enhanced photostability and biocompatibility, improved fluorescent signal (a large number of dye molecules per nanoparticle) and easy conjugation of biomolecules to the nanoparticle surface functional groups [8].

There is growing interest in the fabrication of albumin nanoparticles due to their biocompatibility, biodegradability and non-antigenicity [9,10]. Albumin is one of the most commonly used and characterized proteins in the pharmaceutical field [11]. Soluble albumin and albumin nanoparticles are currently used as a delivery vehicle in chemotherapy as they are known to accumulate and to be catabolized by cancerous tumors [12-14]. NIR dyes such as Indocyanine Green (ICG) and other structurally related cyanine dyes have been shown to have high affinity to albumin [15]. Previous studies also showed that the derivatization of cyanine dyes with carboxylic group/s increases their binding affinity to albumin [16]. Encapsulation of dye molecules within the albumin matrix plays a role in shielding the dye against reactive oxygen species thereby reducing the risk of photobleaching.

**Figure 1:**
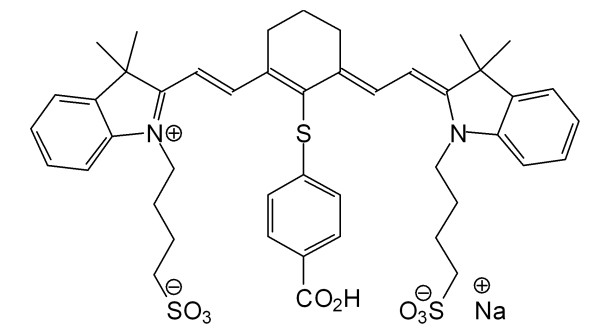
Structure of CANIR dye.

In this work, we have exploited the high affinity of cyanine dyes to albumin for preparation of fluorescent NIR albumin nanoparticles. The dye chosen for use throughout this work is a previously synthesized carboxylic acid derivative of the commercially available NIR dye IR-783 (CANIR, Figure 1). Leakage of the entrapped NIR dye into PBS containing 4% albumin and into human bowel juice was not detected. NIR fluorescent HSA nanoparticles were bioactivated by covalently conjugating targeting agents such as PNA, anti-CEA antibodies (anti-CEA) and anti-TAG-72 antibodies (anti-TAG-72) to the nanoparticle surface. These bioactive NIR fluorescent HSA nanoparticles were found to specifically detect colon cancer tumors, as demonstrated in vivo in a mouse model.

## Results and discussion

**Figure 2:**
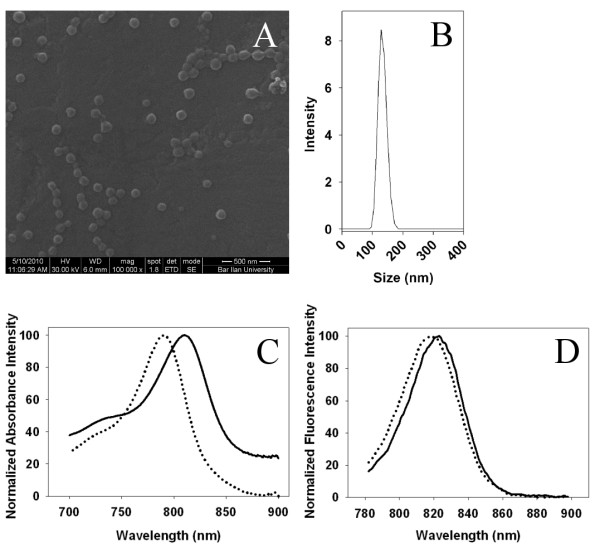
**Characterization of the NIR Fluorescent HSA Nanoparticles. ** (**A,B**) SEM image and hydrodynamic size histogram of the NIR fluorescent HSA nanoparticles; (**C,D**) Normalized absorbance and emission spectra of free CANIR dye (dotted lines) and CANIR-HSA nanoparticles (solid lines), respectively.

Figure2A&B show that the dry and hydrodynamic diameters of the NIR fluorescent HSA nanoparticles used in the present work are 100 ± 15 nm and 140 ± 15 nm, respectively. The hydrodynamic diameter is higher than that of the dry diameter since the hydrodynamic diameter also takes into account the water molecules swollen within the nanoparticles as well as the water molecules adsorbed on its surface [17]. In addition, Figure2C&D illustrates that the maximum absorbance of free CANIR and the NIR fluorescent HSA nanoparticles occurs at approximately 790 and 810 nm, and the maximum fluorescence emission intensity occurs at approximately 818 and 823 nm, respectively. The red-shift of the NIR fluorescent HSA nanoparticles compared to free CANIR dye is probably due to its physical binding to the HSA that affects the dipole moment of the dye. 

Leakage of the encapsulated CANIR dye into PBS dispersion in absence or presence of 4% soluble HSA was not detected following ultrafiltration, as described in the experimental section. There was also no leakage observed when these fluorescent nanoparticles were dispersed in human bowel juice instead of PBS. These results indicate that the CANIR is strongly associated with the albumin nanoparticles, as already known from the literature [18], and the free dye does not leach into the continuous phase (PBS or bowl juice).

### Optimization of the CANIR concentration entrapped within the HSA nanoparticles

In order to optimize the fluorescence intensity of the NIR fluorescent nanoparticles, different concentrations of the CANIR dye were added to the 4% aqueous HSA solution, followed by the formation of the fluorescent nanoparticles via the precipitation process described in the experimental part. The concentration of the entrapped CANIR dye that provided the maximum fluorescence intensity of the NIR fluorescent HSA nanoparticles was 3.0 μg per 0.5 mg particles in 1 mL PBS. At higher dye concentrations, quenching of fluorescence was observed, due to the short distance between the dye molecules entrapped within the nanoparticles, leading to non-emissive energy transfer between them.

### Photobleaching of the NIR fluorescent HSA nanoparticles

**Figure 3:**
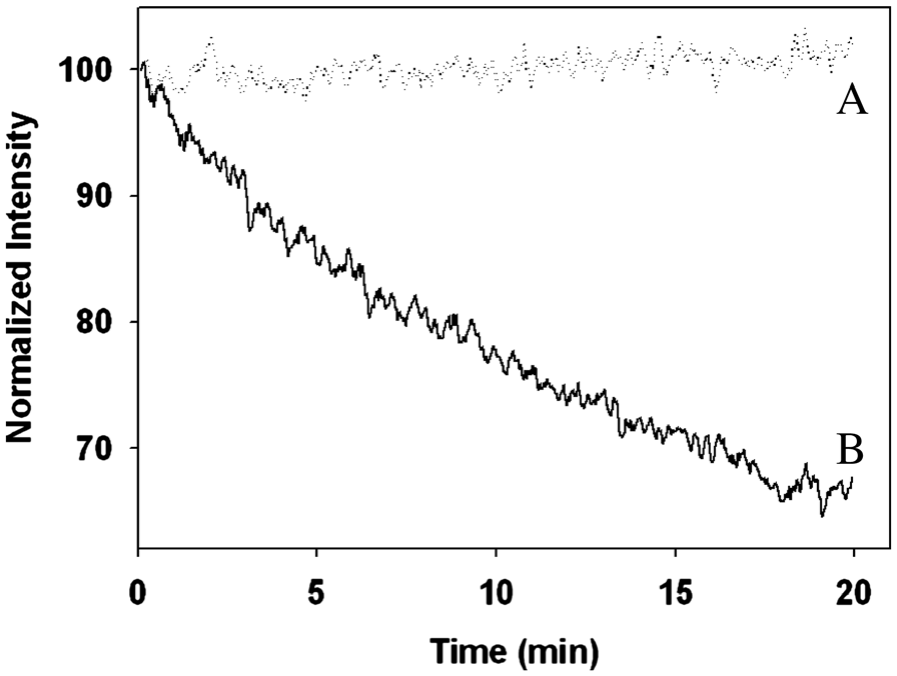
**Photostability of the CANIR-HSA nanoparticles (A) and free CANIR (B) as a function of time.** Samples of CANIR-HSA nanoparticles and free CANIR were illuminated with a xenon flash lamp for 20 min as described in the experimental part.

Photobleaching experiments were performed for free CANIR dye and CANIR encapsulated within the HSA nanoparticles, in order to examine their photostability. Samples of the free CANIR dye and the CANIR entrapped HSA nanoparticles were illuminated at 800 nm and their fluorescence intensities were measured. We have demonstrated that, during illumination, the fluorescence intensity of the NIR fluorescent HSA nanoparticles remains almost unaltered while that of the free CANIR decreases significantly (Figure3), which demonstrates that the encapsulation of the CANIR within the HSA nanoparticles reduces its photobleaching significantly. The encapsulation of the dye probably protects the dye against reactive oxygen species thereby reducing the photobleaching [19]. 

### In vivo optical imaging of human colon tumors in a mouse model

**Figure 4:**
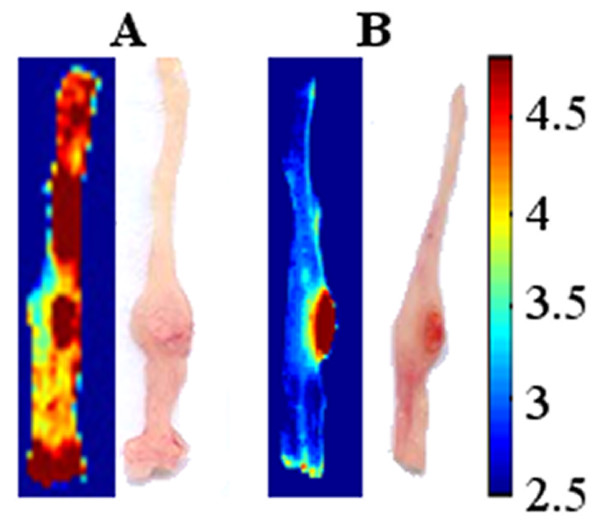
**Color photographs and logarithmically scaled fluorescent images of LS174t tumor cell line from a typical orthotopic mouse model treated with PNA-conjugated NIR fluorescent HSA nanoparticles.** 6 mice were anesthetized and treated with a 0.1% particle dispersion in PBS, via the anus. 20 min later the colons were extensively washed with PBS and were then allowed to recover for 1.5 h (**A**) and for 4 h (**B**). The colons were then removed and treated as described in the experimental part.

For optimization of the tumor detection process, PNA was covalently conjugated to the nanoparticles as described in the experimental part. PNA binds to the terminal sugar β-D-galactosyl-(1–3)-N-acetyl-D-galactosamine of the Thomsen-Friedenreich antigen [20]. In vivo labeling of colonic neoplasms with PNA-conjugated nanoparticles was performed using orthotopic mouse model (6 mice) with colonic tumors originated from LS174t cells injected to the colon wall 2 weeks before the experiment. Mice were anesthetized and treated with a 0.1% PNA-conjugated nanoparticles dispersion in PBS, via the anus. 20 min later the colons were washed extensively with PBS and allowed to recover for 1.5 h (3 mice) and for 4 h (3 mice). The mouse colons were washed, removed and rewashed, as described in the experimental section. A typical fluorescence imaging picture shown in Figure4A exhibits that after 1.5 h of recovery the tumor as well as other parts of the colon were labeled by the fluorescent PNA-conjugated nanoparticles. On the other hand, Figure4B exhibits that after 4 h of recovery the tumors were specifically labeled with a high signal to background ratio (SBR), the “background” referring to the surrounding non-pathological tissue. These results indicate that 4 h of recovery is essential in order to self-wash well the non-specifically adsorbed fluorescent nanoparticles, thereby increasing the SBR. 

Similar mouse trials with 4 h recovery time were performed with targeting agents other than PNA, e.g., monoclonal antibodies against CEA and against TAG-72. For this purpose we conjugated these antibodies to the NIR fluorescent albumin nanoparticles and tested their efficiency for tumor detection in LS174t and HT29 colorectal cancer cell lines. CEA, a highly glycosylated glycoprotein, and TAG-72, a human mucin (MUC1)-like glycoprotein complex, are both highly expressed in most human carcinomas, and therefore used as biomarkers in several modalities of human carcinoma [21-23]. Tumors originating from HT29 cell line serve as a negative control since this cell-line expresses the CEA and TAG-72 antigens to a much lower extent, at least 10^3^ times, lower than the LS174t cell-line [24,25].

**Figure 5:**
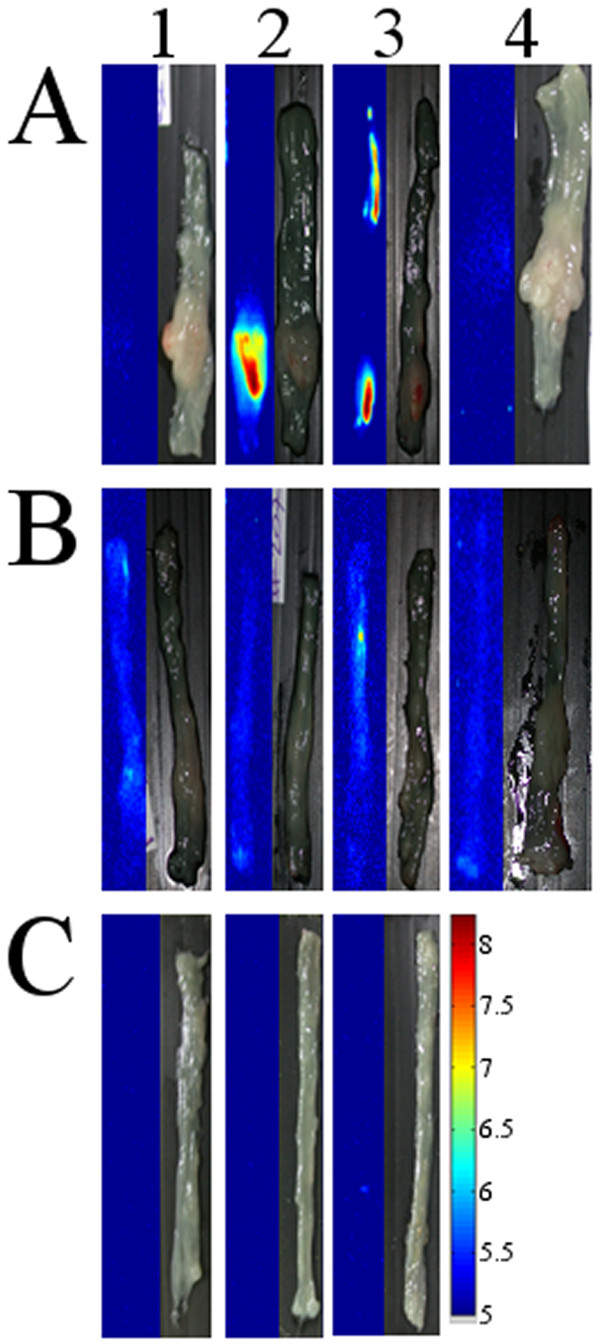
**Logarithmically scaled fluorescent and grayscale images of typical LS174t (A) and HT29 (B) colon tumor cell lines treated with non-conjugated (1) and anti-CEA (2) and anti-TAG-72 (3) conjugated NIR fluorescent HSA nanoparticles: (4) represents untreated tumor cell lines; (C) represents typical colons of healthy mice treated with non-conjugated (1) and anti-CEA (2) and anti-TAG-72 (3) conjugated NIR fluorescent HSA nanoparticles.** 44 mice (each set of experiment was done with 4 mice) were anesthetized and treated with 0.1% particle dispersion in PBS, via the anus. 20 min later the colons were extensively washed with PBS and were then allowed to recover for 4 h. The colons were then removed and treated as described in the experimental part.

Figure5 shows fluorescent and grayscale images of LS174t (A) and HT29 (B) colon tumor cell lines treated with non-conjugated (1) and anti-CEA (2) and anti-TAG-72 (3) conjugated NIR fluorescent HSA nanoparticles. This Figure clearly illustrates that the non-conjugated nanoparticles did not detect the tumors of both LS174t and HT29 cell lines. On the other hand, the anti-CEA and anti-TAG-72-conjugated nanoparticles selectively labeled the LS174t tumor with very high SBR (40–50), but did not significantly label the HT29 tumors, as expected. These results are probably due to the significant difference in the concentration of the CEA and TAG-72 receptors on these cell lines, as mentioned previously. In addition, Figure5C illustrates that the anti-CEA and anti-TAG-72-conjugated NIR fluorescent HSA nanoparticles did not label the colons of the healthy mice. Also, Figure 5A4 and 5B4 indicate that under the experimental conditions the autofluorescence signal of the non-treated tumor cell lines is negligible.

We should also note that the mouse colon treated with the anti-TAG-72-conjugated fluorescent HSA nanoparticles shown in Figure 5A3 has two clear fluorescent signals (both with a SBR of approximately 50). The tumor in the distal part of the colon is visible to the eye, whereas the more proximal fluorescent signal was only revealed as pathological subsequent to histological analysis. This result may suggest a significant advantage of NIR fluorescence imaging using NIR fluorescent nanoparticles, over regular colonoscopy.

**Figure 6:**
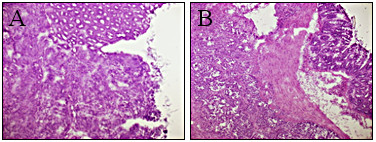
Histological analysis (H&E staining) of lumen-facing (A) and non-lumen-facing (B) tumors treated with anti-CEA-conjugated NIR fluorescent HSA nanoparticles.

It should be noted that most tumors developed in the mouse colons in the orthotopic model used were exposed to the colon lumen. However, infrequently tumors grew in the submucosa and were not exposed the colon lumen. Figure6 exhibits for example the histological analysis of lumen-exposed (Figure6A) and lumen-not exposed (Figure6B) tumors. It seems that our biomolecule-conjugated NIR fluorescent HSA nanoparticles detect specifically the tumors exposed to the lumen and not those not exposed to the colon lumen.

**Figure 7:**
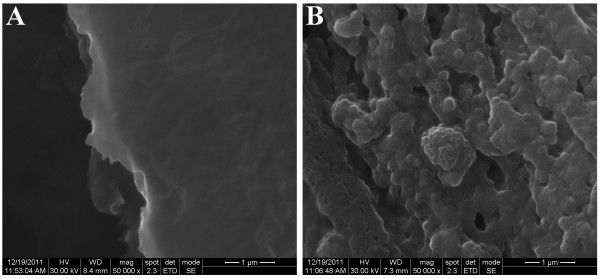
SEM images of sections from LS174t tumor implants of a mouse colon untreated (A) and treated (B) with the anti-CEA-conjugated NIR fluorescent HSA nanoparticles.

In order to prove that the bioactivated NIR fluorescent HSA nanoparticles are indeed responsible for the fluorescent signal observed, tumor sections were also analyzed by SEM as shown in Figure7. This figure illustrates that the section belonging to the untreated tumor does not contain nanoparticles (Figure7A), whereas the treated tumors contain observable nanoparticles.

## Conclusions

The NIR fluorescent HSA nanoparticles discussed in this work have the potential to assist in early diagnosis of colonic neoplasms. The PNA, anti-CEA and anti-TAG-72-conjugated nanoparticles specifically label in vivo the mice colon tumor tissues. While detection of cancer by these bioactivated nanoparticles has been demonstrated, the conjugation of a few targeting agents is likely to significantly enhance the fluorescent signal and the specificity, and therefore enhance their efficiency as diagnostic biomaterials.

In future work, the particles could be used for in vivo detection using either whole body fluorescence imaging or a newly developed fluorescent endoscopy camera. We plan to extend this study to include additional tumor-targeting ligands including antibodies, proteins and small peptides such as epidermal growth factor (EGF) and TRAIL [26,27]. In addition, cancer drugs such as paclitaxel and doxorubicin may also be encapsulated within these NIR fluorescent HSA nanoparticles, so that these nanoparticles may be used for both diagnosis and therapy.

## Materials and methods

### Materials

The following analytical-grade chemicals were purchased from commercial sources and were used without further purification: 2-[2-[2-Chloro-3-[2-[1,3-dihydro-3,3-dimethyl-1-(4-sulfobutyl)-2 H-indol-2-ylidene]ethylidene]-1 cyclohexen-1-yl]ethenyl]-3,3-dimethyl-1-(4-sulfobutyl)-3 H-indolium inner salt sodium salt (IR-783), HSA, 4-mercaptobenzoic acid, *N,N*-dimethylformamide (DMF), 1-ethyl-3-(3-dimethylaminopropyl) carbodiimide (EDC), sepharose 4B, peanut agglutinin (PNA) and fluorescein isothiocyanate-conjugated peanut agglutinin (FITC-PNA), hematoxylin and eosin from Sigma (Rehovot, Israel); *N*-hydroxysulfosuccinimide (Sulfo-NHS) from Thermo Scientific, U.S.A; 2-morpholino ethanesulfonic acid (MES, pH 6) from Fisher Scientific, U.S.A; Tissue-Tek® O.C.T. from Sakura, Japan. Phosphate Buffered Saline (PBS), Minimum Essential Medium (MEM) eagle, McCoy's 5A medium and Dulbecco's modification of eagle's medium (DMEM), glutamine, penicillin and streptomycin from Bet Haemek, Israel; LS174t and HT29 tumor cell lines from American Type Culture Collection (ATCC); monoclonal anti-CEA (T86-66) and anti-TAG-72 (CC-49) antibodies were purified from the hybridoma supernatant at Alomone Labs, Israel; human bowel juice were obtained from Prof. A. Nissan, Surgery Department, Rabin Medical Center, Beilinson Hospital, Israel. Water was purified by passing deionized water through an Elgastat Spectrum reverse osmosis system (Elga Ltd., High Wycombe, UK).

### Synthesis of the NIR fluorescent CANIR dye

CANIR (Figure1) was prepared by interacting the commercially available IR-783 with 4-mercaptobenzoic acid, as described by Strekowski et al. [28]. Briefly, IR-783 (250 mg, 0.33 mmol) and 4-mercaptobenzoic acid (154 mg, 1 mmol) were dissolved in 10 mL DMF. This solution was stirred overnight at room temperature. A mixture of EtOH/ether (1:20, 20 mL) was added dropwise and the dark precipitate was separated by centrifugation, washed with ether and lyophilized. The structure and purity (99%) was confirmed by elemental analysis, MS and ^1^ H and ^13^ C NMR. 

### Synthesis of the NIR fluorescent HSA nanoparticles

The NIR fluorescent HSA nanoparticles were prepared by a desolvation technique via a precipitation process [29,30]. Briefly, ethanol (12.5 mL) was added to an aqueous solution (6.25 mL) of HSA (250 mg) and CANIR (1.25 mg). The obtained solution was then stirred at room temperature for 15 min at 800 rpm. Then, the temperature was raised to 70°C and the mixture was stirred for additional 4 h, followed by 78°C for 12 h. The obtained NIR fluorescent particles dispersed in water were then washed from impurities by sepharose 4B gel filtration.

Non-fluorescent HSA nanoparticles were prepared similarly to those of the NIR fluorescent HSA nanoparticles, in the absence of the CANIR dye.

### Determination of the encapsulated CANIR concentration in the NIR fluorescent HSA nanoparticles

The concentration of the encapsulated CANIR was determined for 0.5 mg/mL nanoparticles, via a calibration curve obtained by measuring the integral of absorbance peaks of standard solutions of free CANIR in PBS (at wavelengths 630 – 900 nm).

### Optimization of the CANIR concentration encapsulated within the HSA nanoparticles

Different concentrations of the CANIR dye were added to the 4% aqueous HSA solution (weight % ratio of the CANIR dye and the NIR fluorescent HSA nanoparticles were 0.1, 0.25, 0.5, 0.7 and 1), followed by the formation of the fluorescent nanoparticles. The NIR fluorescent HSA nanoparticles dispersed in PBS were diluted with PBS to 0.5 mg/mL and their fluorescence intensities at 823 nm were then measured.

### Leakage extent of the CANIR encapsulated in the fluorescent HSA nanoparticles into PBS and human bowel juice

4% HSA solution in PBS containing the NIR fluorescent HSA nanoparticle (1 mg/mL) was shaken at 37°C for 4 h and then filtered via a 300-kDa filtration tube (VS0241 VIVA SPIN) at 4000 rpm. The fluorescence intensity of the supernatant was then measured at 750 nm. A similar procedure was performed with human bowel juice as a continuous phase substituting the 4% HSA solution in PBS.

### Conjugation of the tumor-targeting ligands (PNA, anti-CEA and anti-TAG-72) to the NIR fluorescent HSA nanoparticles

PNA was covalently conjugated to the nanoparticle surface carboxylate groups via the cabodiimide activation method [31]. Briefly, EDC (1 mg) and Sulfo-NHS (1 mg) were each dissolved in 0.1 M MES (pH 6.0, 1 mL) containing 0.5 M NaCl. The EDC solution (1 mg/mL, 10 μL) was added to an aqueous solution of PNA (0.25 mg, 62.5 μL), followed by the addition of the sulfo-NHS solution (1 mg/mL, 25 μL). The mixture was then shaken at room temperature for 15 min, followed by the addition of the NIR fluorescent HSA nanoparticles (2.5 mg, 1 mL PBS). The mixture was then shaken for an additional 90 min. The obtained PNA-conjugated fluorescent nanoparticles were then washed from excess reagents by sepharose 4B gel filtration. FITC-PNA, anti-CEA and anti-TAG-72 were conjugated to the NIR fluorescent HSA nanoparticles via a similar procedure substituting the PNA by the other bioactive reagents. The concentration of bound PNA (2.4 μg/mg nanoparticles) was determined with FITC-PNA via a calibration curve of FITC. The concentration of bound anti-CEA and anti-TAG-72 (2.2 ± 0.2 μg/mg nanoparticles) were determined by ELISA.

### Characterization of the NIR fluorescent HSA nanoparticles

Particle size and size distribution were determined by DLS with photon cross-correlation spectroscopy (Nanophox particle analyzer, Sympatec GmbH, Germany) and by Scanning Electron Microscopy (SEM) (JEOL, JSM-840 Model, Japan). For the SEM study, the diameter of more than 100 particles was measured with the image analysis software AnalySIS Auto (Soft Imaging SystemGmbH, Germany).

Fluorescence measurements of the FITC-PNA were performed using a multiplate reader TECAN SpectraFluor Plus (Neotec Scientific Instruments) with excitation wavelength of 485 ± 10 nm and emission wavelength of 535 ± 10 nm, integration time was 40 μs and gain was set to 70.

Fluorescence spectra of the non-conjugated and bioactive conjugated NIR fluorescent HSA nanoparticles were recorded using a spectrofluorometer (Cary eclipse, Agilent Technologies Inc.). Excitation and emission slits were fixed at 5 nm, and λ_ex_ was set at 750 nm. For photobleaching experiments, the samples were diluted to 0.05 M and λ_ex_ was set at 800 nm and λ_em_ at 830 nm. Each of the samples was illuminated continuously, and fluorescence intensity was measured over a period of 20 min. The intensity values were normalized.

### Cell lines

Human colorectal adenocarcinoma cell lines were used for each of the experiments. The LS174t cell line was maintained in Minimum Essential Medium (MEM) eagle supplemented with heat-inactivated FBS (10%), penicillin (100 IU/mL), streptomycin (100 μg/mL) and l-glutamine (2 mM). The HT29 cell line was maintained in McCoy’s 5A medium supplemented with FBS (10%), penicillin (100 IU/mL), streptomycin (100 μg/mL) and l-glutamine (2 mM).

### Detecting of human colon tumor with the non-conjugated and bioactive- conjugated NIR fluorescent HSA nanoparticles in a mouse model

Experiments were performed according to protocols of the Israeli National Council for Animal Experiments by Harlan Biotech, Israel. Cancerous cells (30 uL containing 2x10^6^ LS174t or HT29 cells) were injected into the mouse intestinal wall. 2 weeks later the nude mice (50 mice) were anaesthetized and treated with non-conjugated and bioactive-conjugated NIR fluorescent HSA nanoparticles (0.1%, 200 μL), via the anus, using guidance of a mini-colonoscope. 20 min later each colon was washed with PBS (5 x 1 mL) and mice were allowed to recover for 4 h. The mice were sacrificed and the colons were removed. Each colon was spread on a solid surface, and imaging was performed by using the Odyssey® Infrared Imaging System, (Li-Cor Biosciences, Lincoln, NE, USA) with excitation wavelength 780 nm and emission wavelength 800 nm.

### Histological analysis

Following the in vivo mice experiments the tissues were frozen in liquid nitrogen in Tissue-Tek® O.C.T. compound and cryosectioned at 5 microns in the cryostat. The sections were picked up on a glass slide and stained with hematoxylin and eosin (H&E) for histopathological examination. The sections were also spread on a stab, stained with H&E and visualized by SEM.

## Competing interests

The authors declare that they have no competing interests.

## Authors' contributions

SC carried out the synthesis and characterization of the nanoparticles. SM supervized the study, and participated in its design and coordination. Both authors read and approved the final manuscript.
